# Exploring Influenza Vaccine Uptake and Its Determinants among University Students: A Cross-Sectional Study

**DOI:** 10.3390/vaccines8010052

**Published:** 2020-01-28

**Authors:** Yukako Kawahara, Hiroshi Nishiura

**Affiliations:** 1 Graduate School of Medicine, Hokkaido University, Kita 15 Jo Nishi 7 Chome, Kita-ku, Sapporo-shi, Hokkaido 060-8638, Japan; ykawahara@academic.hokudai.ac.jp; 2 Hokkaido University Health Center, Kita 16 Jo Nishi 7 Chome, Kita-ku, Sapporo-shi, Hokkaido 060-0816, Japan

**Keywords:** immunization, prevention, epidemiology, students, vaccination effectiveness

## Abstract

While vaccination is the only established option to prevent a susceptible host from influenza, we have yet to clarify the decision-making mechanisms of vaccine uptake among Japanese university and college students. We aimed to explore vaccination coverage and the related demographic, sociocultural, and socioeconomic factors among university students. We performed a cross-sectional survey involving 604 students at Hokkaido University. Participants were asked if they received influenza vaccination in advance of the 2018/19 season, and subsequently, their demographic and sociocultural/socioeconomic characteristics were surveyed. We also explored the mechanisms underlying students’ vaccination decisions. Vaccination coverage was estimated at 27.3% (95% confidence interval: 23.7–30.9). Freshmen (*p* < 0.0001) were significantly associated with choosing vaccination, and their odds ratio of vaccination was 11.3 (95% confidence interval: 6.2–20.7) times greater than students in other years. Among students other than freshmen, students belonging to medicine- and healthcare-related faculties were vaccinated three times more frequently than other students, and the coverage in students from Hokkaido was twice as large as that for students from other prefectures. Moreover, extracurricular activity was a positive predictor of vaccination. Although the coverage was as small as 27.3% among university students, freshmen in Japan have high vaccination coverage, which we believe is associated with the entrance examination during high influenza activity. In addition to exposing students to proper education regarding their risk self-assessment, consciousness raising via appropriate understanding of influenza and its severity and offering vaccination at university health centers at a reasonable cost may promote vaccine acceptance.

## 1. Introduction

Influenza is a viral infectious disease caused by the influenza virus, an RNA virus that belongs to the family Orthomyxoviridae [1]. Seasonal influenza epidemics occur in winter to early spring. Influenza is distinguished from the common cold by a fever of >38.0 °C that is frequently accompanied by upper respiratory symptoms of sore throat, cough, and nasal discharge, and also by chills, headache, and muscular and joint pain [2]. Annually, influenza is responsible for approximately 291,000–646,000 deaths worldwide [3,4], and the social and economic impact includes not only absenteeism and the need to seek medical attention, but also restricted range of movement during the symptomatic period and loss of work [5,6,7]. 

Vaccination is the only established option to prevent a susceptible host from contracting influenza infection and developing its severe complications [8,9]. Immunization offers direct protection in vaccinated individuals as well as “herd immunity” to the population via indirect reduction in the opportunities for infection [10,11,12,13,14,15]. In many countries, influenza vaccination is part of a national immunization program, and Japan is no exception. However, the routine immunization program in Japan, which rests on Immunization Law and is conducted by local government, covers only older people with underlying comorbidities such as chronic obstructive pulmonary disease and chronic renal failure, and vaccination in other age groups, including children, is voluntary. 

University and college students in Japan are known as not achieving high vaccination coverage, which is estimated at 20%–30% [16,17,18,19], and coverage surveys have indicated that younger school children and older adults achieve higher coverage at a rate of 50% for each group [20,21,22,23], which is not substantial enough to establish herd immunity [24]. Influenza among university and college students results in absenteeism of at least 5 days because of the time from illness onset and at least 2 days required to recover from pyrexia. Moreover, the total cost required for the medical services (i.e., physical examination, rapid diagnostic testing using nasopharyngeal samples, and prescription of antivirals) for a single student is 10,000 Japanese Yen (equivalent to 90 US dollars as of November 2019) [25,26,27]. Considering that students belong to various communities and are highly mobile both domestically and internationally [10,13,16,23,27], their vaccination is of utmost importance to prevent the spread of the disease within the community.

While the determinants of vaccination were explored among working-age people in Japan [28,29,30], studies have yet to clarify what demographic, sociocultural, and socioeconomic factors characterize university and college students’ intentions to undertake vaccination. In other countries, university and college students’ vaccination has been surveyed more closely [31,32,33,34]. For instance, in the USA, vaccination coverage among medical students appeared to be low, at approximately 40%–60% [35,36,37,38]. In other countries and levels of expertise, the coverage varies, with nursing students at 15.2% in Hong Kong [39], medical students in China at <10% [40], medical students in Australia at 30%–50% [41], and medical students in Italy at 54% [42]. To explain the variation, students’ socioeconomic class was associated with vaccine uptake [43,44,45], and students’ attitudes toward vaccination (e.g., safety concerns regarding adverse events) was shown to influence vaccination uptake among nursing and public health students [35,36,40,46]. In Japan, the decision-making mechanisms in vaccine uptake in Japanese university and college students have not been clarified, which prevents objectively designing strategies to prevent influenza via vaccination. The present study aimed to explore the vaccination coverage and its demographic, sociocultural, and socioeconomic factors among university students. 

## 2. Materials and Methods 

### 2.1. Cross-Sectional Survey

We performed a questionnaire-based cross-sectional survey to identify the epidemiological determinants of vaccine uptake among students to help improve vaccination programs. We conducted the survey at Hokkaido University during students’ annual health check-ups, organized by the University Health Center, in April 2019. In Japan, all university students are mandated to undertake general health check-ups according to the School Health and Safety Act. The first author performed the in-person questionnaire surveys at the health check-up zone on campus and also at the entrance to the canteen during the above-mentioned period. Before students completed the survey, the first author explained the purpose and contents of the survey, and only when students agreed to participate, were they asked to answer questions on a single sheet of paper (see Additional File 1), which required approximately 5–10 min per person. We used convenience sampling, and of the total of 11,210 students, we invited 604 students because we assumed that vaccination coverage was approximately 17% [11,12,13], and we aimed to detect any difference with a reliability of 95% and error of 3%.

### 2.2. Questionnaire Survey

We used a self-administered non-standardized questionnaire survey, in this study. Participants were asked if they received influenza vaccination in advance of the 2018/19 season. We recorded participants’ demographic variables, namely, sex, age, grade/year, academic major (faculty), place of birth (Hokkaido or elsewhere), and whether they engaged in extracurricular activities (e.g., sports club). To understand answering categories, Table S1 (Supplementary Material) shows the English translation of our questionnaire. Regarding the socioeconomic variables that potentially explained the mechanisms underlying the decision to receive vaccination, we asked (i) whether participants received a monthly allowance from their parents, (ii) whether participants received a scholarship, and (iii) students’ parental education levels (e.g., up to high school or university or postgraduate level). We also asked participants about the mechanisms underlying their vaccination decision; participants checked the following item if it applied: (a) worried about the risk of contracting influenza, (b) dislike injections (because of pain), (c) worried about the clinical seriousness of influenza, (d) a perception of limited vaccine effectiveness, (e) concerned with the cost of vaccination, (f) worried about having an allergic reaction to a vaccine, (g) would decide depending on the vaccination status of the surrounding people, (h) would depend on the availability of a nearby healthcare expert, (i) worried about adverse reactions following vaccination, (j) whether their general health status is good, and (k) whether students had sufficient time to undertake vaccination. The questionnaire was anonymously handled, and its electronical input was made by the first author, followed by a double-check by the second author.

### 2.3. Statistical Analysis

First, we examined participants’ descriptive characteristics, including their demographic and socioeconomic aspects. We estimated the overall vaccination coverage at Hokkaido University, as the main outcome. Second, we performed a univariate analysis to analyze the association between influenza vaccination and each possible explanatory variable. Because both vaccination and explanatory variables were considered dichotomous, we used the chi-square or Fisher’s exact test. We then calculated the odds ratio (OR) of vaccination for a given status of the explanatory variable. Third, cross-tabulation of vaccination coverage by two variables that were significantly associated with vaccination during univariate analysis was explored to examine possible interactions. Finally, maintaining vaccination coverage as the outcome, we investigated students’ attitudes toward influenza vaccination that could lead to a vaccination decision. Students’ attitude was considered a dichotomous outcome, and we used the chi-square or Fisher’s exact test and calculated ORs. *p* < 0.05 was considered statistically significant.

### 2.4. Ethical Concerns

Prior to the beginning of the questionnaire survey, students were informed of the study purpose, and written informed consent was obtained. The first author explained how the information would be used and assured students of the confidentiality of their responses. The research team explained that enrollment in this study was voluntary, and gave students the explicit right to withdraw at any time. When the student was younger than 20 years of age, the student’s parent or guardian was asked to sign the consent form on his/her behalf and given the chance to decline to sign the document. The Medical Ethics Committee of Hokkaido University Graduate School of Medicine (Japan) reviewed and approved this study (ID: Med19-002).

## 3. Results

Of the 11,266 students at Hokkaido University as of April 2019, 604 students (5.4%) responded to the questionnaire. Excluding graduate students, students from other universities, and those with difficulty reading Japanese (and not enrolled in faculty programs), data from 593 students were eligible for subsequent statistical analyses. Table 1 summarizes the distributions of the surveyed variables, namely, sex, age, and grade/year, compared with data for the entire university. Participants’ sex, grade/year, faculty, and place of birth did not differ from data for the entire population. A total of 162 students received vaccination in the 2018/19 season, and the vaccination coverage was estimated at 27.3% (95% confidence interval (CI): 23.7–30.9).

Table 2 summarizes the univariate relationship between influenza vaccine uptake and the surveyed variables. Younger age (*p* < 0.0001) and being a freshman (*p* < 0.0001) were significantly associated with vaccine uptake, and the OR of vaccination among these groups was 2.7 (95% CI: 1.9–3.9) and 11.3 (95% CI: 6.2–20.7) times greater, respectively, than for remainders. Students who belonged to the Faculty of Medicine or who were involved in other healthcare faculties (i.e., Health, Pharmacy, and Dentistry) were more likely to be vaccinated than students in other faculties (OR = 2.5; 95% CI: 1.5–4.1; *p* < 0.001), and students born in Hokkaido were also more likely to be vaccinated (OR = 1.8; 95% CI: 1.2, 2.7; *p* < 0.0001). Students engaging in extracurricular activities were less likely to be vaccinated than students not engaging in extracurricular activities (OR = 0.3; 95% CI: 0.2–0.5); *p* < 0.0001). Sex, monthly allowance, scholarship, and parental education levels were not associated with vaccine uptake.

Table 3 shows the cross-tabulations of the variables that were significantly associated with vaccination during the univariate analysis, with special focus on freshman or not as an explanatory variable. Stratifying grade/year by age (i.e., 18 and 19 years, or ≥20 years), freshmen were more frequently vaccinated than other year levels (OR = 11.4; 95% CI: 5.4–23.9; *p* < 0.0001) among 18- and 19-year-old students, and no significant association was identified for other cross-tabulated comparisons using age and grade/year. This indicated that age was not associated with vaccination, and whether a student was a freshman among 18- and 19-year-old students determined the vaccination decision. Similarly, when we stratified vaccination coverage by grade/year and place of birth, being born in Hokkaido was positively associated with vaccination only among non-freshman (OR = 2.1; 95% 1.4–3.3; *p* = 0.0012). Being a freshman was always positively associated with vaccine uptake (*p* < 0.001) for people from Hokkaido and elsewhere. Stratifying by grade/year and extracurricular activity, extracurricular activity was negatively associated with vaccination (*p* = 0.01), but the association was opposite among those not engaging in extracurricular activities (*p* = 0.02). Again, being a freshman was always positively associated with vaccine uptake for both engaging in and not engaging in extracurricular activities.

Table 4 shows the cross-tabulations of the variables that were significantly associated with vaccination during the univariate analysis, with special focus on medicine and healthcare faculties or other faculty as an explanatory variable. Regardless of whether a student was in a medicine or healthcare faculty, being a freshman was a predictor of vaccination (*p* = 0.04 vs. < 0.0001, respectively, for medicine and healthcare vs. other faculties). Being a non-freshmen in medicine and healthcare faculties was a positive predictor of vaccination (*p* < 0.0001; OR = 3.1; 95% CI: 1.8–5.3). Students from Hokkaido were more likely to be vaccinated only among those not studying medicine or healthcare (*p* < 0.03; OR = 1.7; 95% CI: 1.0–2.6), and among students raised in locations other than Hokkaido, students in medicine and healthcare were more likely to be vaccinated than students in other faculties (*p* = 0.01; OR = 2.5 (95% CI: 1.3–4.8)). Among students other than those in medicine and healthcare, engaging in extracurricular activities was a negative predictor of vaccination (*p* < 0.0001; OR = 0.2; 95% CI: 0.1–0.4), and among students engaging in extracurricular activities, students in medicine and healthcare were more likely to be vaccinated than those in other faculties (*p* = 0.0002; OR = 2.9; 95% CI: 1.7–5.0).

Table 5 summarizes students’ attitudes toward influenza and influenza vaccination. Four notable negative predictors were identified, namely, (i) not worried about the risk of infection (*p* = 0.04; OR = 0.7; 95% CI: 0.4–1.0), (ii) dislike injections or injections are painful (*p* = 0.01; OR = 0.2; 95% CI: 0.1–0.8), (iii) not worried about the clinical seriousness of influenza (*p* = 0.005; OR = 0.4; 95% CI: 0.2–0.8), and (iv) concerned with the cost of the influenza vaccine (*p* = 0.004; OR = 0.6; 95% CI: 0.4–0.8). Among the other potential explanations for not choosing vaccination, limited effectiveness, vaccination status of the surrounding people, potential adverse reaction, parental advice, and available time were not associated with receiving influenza vaccination.

## 4. Discussion

This study was a cross-sectional survey of influenza vaccination history among Hokkaido University students, which identified a vaccination coverage rate of 27.3% among approximately 600 students. While the overall vaccination coverage could be affected by the year of the study, univariate and cross-tabulation analyses showed that being a freshman was a strong positive predictor of vaccination, and that freshmen were approximately 10 times more frequently vaccinated than students in other years. Among non-freshmen, students belonging to medicine and healthcare-related faculties were three times more frequently vaccinated than students in other faculties, and the coverage of students raised in Hokkaido was twice as high as that for students from other locales. Moreover, engaging in extracurricular activities was a positive predictor of vaccination among students other than freshmen. Regarding students’ attitudes toward influenza vaccination, students paying little attention to the risks of infection and the severity of influenza and those concerned about injection-related pain and vaccination cost were less likely to be vaccinated. To our knowledge, our study is the first to explore the demographic and sociocultural factors related to influenza vaccination in a university setting in Japan with students enrolled in diverse disciplines, including natural and social sciences. 

A remarkable finding in the present study was the high vaccination coverage (75.4%) among freshmen who completed the entrance examination for Hokkaido University from February to March, 2019 (i.e., 1–2 months prior to the survey). In Japan, there is a very competitive process to enter high-ranking universities [47,48], and considering that high school students are explicitly advised to undertake influenza vaccination, we believe that high school students’ decisions for vaccination were associated with the entrance examination, which takes place in the winter season during high influenza activity. A relatively high vaccination coverage of 31.5% among freshmen was also reported from the USA [49]. Of course, parents could have encouraged students to be vaccinated in advance of their own decision. In addition to the high coverage in freshman, students in medicine and healthcare faculties were more likely to be vaccinated among non-freshman (40.3%), perhaps reflecting their potentially higher exposure at healthcare facilities. Belonging to medicine and healthcare faculties was not associated with vaccination among freshman, because many freshmen do not belong to the medicine and healthcare faculties in their first year, but can choose to proceed to these faculties in their second year. It is also true that freshmen in medicine and healthcare faculties have very little chance to interact with patients, not motivating them for vaccination. The estimated coverage among students in medicine and healthcare faculties was lower but comparable to that in Australia (53.8%) [41] and similar to the rate in Southern California (43.0%) [35]. Making the vaccination compulsory increases vaccination coverage among healthcare students [39,41]. These determinants of vaccination i.e., freshman and medical student, are not translatable to all related studies, but our findings were similar to those in previous studies [35,41,47,48,49].

Importantly, we also found that students other than freshmen were more likely to be vaccinated if they were from Hokkaido (31.0%) and if they engaged in extracurricular activities (22.2%). The majority of students from Hokkaido live with their parents and commute to the university, or at least have frequent chances to visit their homes on the weekends. Economically stable living conditions and continued exposure to health-related input from their family members could have contributed to the elevated vaccination coverage. Extracurricular activity increases the chance to interact with other students; however, this activity was not associated with freshmen because they have yet to decide on an extracurricular activity. As implied by social epidemiological studies [19,50,51,52], the level of social capital promotes healthy decision-making. In particular, students engaging in social interactions via extracurricular activities, including extracurricular campus activities and volunteering in local community service, have higher vaccination coverage rates [34]. Educating students regarding their risk self-assessment may promote vaccination [37].

Unvaccinated participants provided their reasons for considering or not considering vaccination, including injection pain, perceived risk of influenza and its severe complications, and the cost of vaccination. Appropriate education from university healthcare workers could temporarily eliminate these concerns, but education alone does not directly lead to behavioral change toward vaccination [19,35,37,53]. When devising an appropriate health recognition model for students, it is essential to develop diverse opportunities for communication at home, in extracurricular activities, and other lifestyle environments on-campus [31,32,34]. There are four different transtheoretical models/components for behavioral change, i.e., (i) balancing the advantages vs. disadvantages of change, (ii) ensuring self-efficacy, (iii) promoting and supporting experiential and behavioral processes, and (iv) accounting for stages of change [54,55,56,57,58]. In our setting, (i) balancing advantages and disadvantages could affect the results. Consciousness raising and relieving students’ concerns could be attained by understanding that influenza can be fatal, and possible consideration of vaccination at the university health center at low cost could lead to environmental reevaluation. Although a radical option, (ii) mandating compulsory vaccination for all students could act as a counter-conditioning factor as part of (iii) the process of behavioral change. Such a program could potentially be aligned with the annual health check-up.

Strengths of our study include (i) a balanced involvement of both freshmen and others via questionnaire survey at the very beginning of an academic year, (ii) the study at a single university where all faculty buildings are built on the same campus, and (iii) good representation of the entire university student body including accurate sex ratio representation. Limitations of the study must be discussed. First, this study had a cross-sectional design, and thus, temporality was not assessed, and causal relationships remain unknown. Second, potential systematic biases of our study include (i) responder selection bias (e.g., those accepting our survey were likely to have been vaccinated) and (ii) interviewer bias (e.g., students who were more accessible to the authors could have shared some propensity of vaccination tendency. Both could have elevated the vaccination coverage. Although we did not explicitly address confounders, we at least examined potential interaction for each combination of two variables. Third, we performed this survey in April 2019, the first month of the academic year in Japan. For this reason, the extracurricular activities and future majors (and faculties) of the freshmen remained largely unknown, making these items difficult to assess. Fourth, while we hypothesized that the most important reason for vaccination among freshmen was to avoid influenza during the entrance examination, we missed the chance to ask whether students would be keen to receive vaccination for some other event, especially important events associated with university life. Fifth, the sample size was limited to approximately 600, and we were unable to identify more precise characteristics (e.g., vaccination coverage of a specific faculty other than medicine and healthcare). Sixth, the questionnaire could have been revised to explore the possible contributors to behavioral change (e.g., factors associated with attitudes and decision-making processes) based on transtheoretical models. 

Despite these limitations, we believe that we have successfully shown that an important life event (e.g., entrance examination) can enormously elevate vaccination coverage. In the future, university curriculum designs could account for our finding and organize key events during the winter season. Also, considering the concerns over the cost of vaccination, an aggressive option is to implement compulsory vaccination free of charge, perhaps starting it among medicine and healthcare students. In addition to providing students with the tools to assess their own risk, consciousness raising via appropriate understanding of influenza and its severity and offering vaccination at university health centers at a reasonable cost would promote vaccine acceptance.

## 5. Conclusions

The present study involved a cross-sectional survey of influenza vaccination history among Hokkaido University students. The vaccination coverage was 27.3%, and freshmen were approximately 10 times more frequently vaccinated than students in other years. Among students other than freshmen, belonging to medicine and healthcare faculties, engaging in extracurricular activities, and being raised in Hokkaido were positive predictors of vaccination. Consciousness raising via appropriate understanding of influenza and its severity and offering vaccination at university health centers at a reasonable cost would promote vaccine acceptance.

## Figures and Tables

**Table 1:** Characteristics of the study participants (*n* = 593).

Variable	Participants (%) ^†^	Hokkaido University Students (%)
**Sex**		
Male	401 (68.0)	8032 (71.3)
Female	190 (32.0)	3234 (28.7)
**Age**		
18 and 19 years	197 (33.6)	NA
≥20 years	390 (66.4)	NA
**Grade/Year**		
Freshman	65 (11.0)	2668 (24.0)
Other years	527 (88.9)	8598 (76.0)
**Faculty**		
Medicine and Healthcare *	56 (10.0)	1126 (10.0)
Other	504 (90.0)	10 140 (90.0)
**Place of birth**		
Hokkaido	164 (27.8)	816 (32.0)
Elsewhere	427 (72.3)	1748 (68.0)
**Monthly allowance from parents**		
Received	402 (81.2)	NA
Not received	93 (18.8)	NA
**Scholarship**		
Received	117 (31.1)	NA
Not received	259 (68.9)	NA
**Parental education (father)**		
High school level or lower	139 (24.0)	NA
University level or higher	441 (76.0)	NA
**Parental education (mother)**		
High school level or lower	226 (38.8)	NA
University level or higher	356 (61.2)	NA
**Extracurricular activity**		
Yes	519 (88.4)	NA
No	68 (11.6)	NA
**Vaccination status**		
Vaccinated	162 (27.3)	NA
Unvaccinated	431 (72.3)	NA

NA = data not available; * Medicine and Healthcare indicates the faculties of Medicine, Health, Pharmacy, and Dentistry; † the sample size of the participants was 593, but for each item there were a small number of missing answers, resulting in <593 answers.

**Table 2:** Frequency of influenza vaccination according to Hokkaido University students’ characteristics.

Variable	Vaccinated (%)	Unvaccinated (%)	*p*-Value	OR* (95% Cl**)
**Sex**				
Male	107 (26.7)	294 (73.3)		
Female	54 (28.4)	136 (71.6)	0.66	0.9 (0.6–1.3)
**Age**				
18 and 19 years	81 (41.1)	116 (58.9)		
≥20 years	80 (20.5)	310 (79.5)	<0.0001	2.7 (1.9–3.9)
**Grade/Year**				
Freshman	49 (75.4)	16 (24.6)		
Other years	112 (21.3)	415 (78.8)	<0.0001	11.3 (6.2–20.7)
**Faculty**				
Medicine and Healthcare *	35 (44.3)	44 (55.7)		
Other faculties	116 (24.1)	365 (75.9)	<0.0001	2.5 (1.5–4.1)
**Place of birth**				
Hokkaido	60 (36.6)	104 (63.4)		
Elsewhere	102 (23.9)	325 (76.1)	<0.0001	1.8 (1.2–2.7)
**Monthly allowance from parents**				
Received	102 (25.4)	300 (74.6)		
Not received	27 (29.0)	66 (71.0)	0.47	0.8 (0.5–1.4)
**Scholarship**				
Received	30 (25.6)	87 (74.4)		
Not received	72 (27.8)	187 (72.2)	0.66	0.9 (0.5–1.5)
**Parental education (father)**				
High school level or lower	38 (27.3)	101 (72.5)		
University level or higher	116 (26.3)	325 (73.7)	0.81	1.1 (0.7–1.6)
**Parental education (mother)**				
High school level or lower	60 (26.6)	166 (73.5)		
University level or higher	96 (27.0)	260 (73.0)	0.91	1.0 (0.7–1.4)
**Extracurricular activity**				
Yes	121 (23.3)	398 (76.7)		
No	36 (52.9)	32 (47.1)	<0.0001	0.3 (0.2–0.5)

*p*-value, chi-square test; * OR = odds ratio of being vaccinated if a student was in the category on the first line vs. the second line (e.g., men’s odds ratio of vaccination compared with that of women); ** Cl = confidence interval using Wald’s method.

**Table 3:** Cross tabulation of the frequency of vaccination, regarding age, grade/year, birthplace, and extracurricular activity.

Characteristic	N (%)	Vaccinated (%)	Unvaccinated (%)	*p*-Value	OR * (95% Cl **)
**Age**					
Freshman					
18 and 19 years	58 (89.2)	46 (79.3)	12 (20.7)	0.06	5.1 (1.0–26.0)
≥20 years	7 (10.8)	3 (42.9)	4 (57.1)		
Other ages					
18 and 19 years	139 (26.7)	35 (25.2)	104 (74.8)	0.23	1.4 (0.9–2.1)
≥20 years	382 (73.3)	76 (19.9)	306 (80.1)		
18 and 19 years					
Freshman	58 (29.4)	46 (79.3)	12 (20.7)	<0.0001	11.4 (5.4–23.9)
Other years	139 (70.6)	35 (25.2)	104 (74.8)		
≥20 years					
Freshman	7 (1.8)	3 (42.9)	4 (57.1)	0.15	3.0 (0.7–13.8)
Other years	382 (98.2)	76 (19.9)	306 (80.1)		
**Place of birth**					
Freshman					
Hokkaido	19 (30.0)	15 (79.0)	4 (21.1)	1.00	1.2 (0.3–4.4)
Elsewhere	45 (70.3)	34 (75.6)	11 (24.4)		
Other years					
Hokkaido	145 (27.6)	45 (31.0)	100 (69.0)	0.0012	2.1 (1.4–3.3)
Elsewhere	381 (72.4)	67 (17.6)	314 (82.4)		
Hokkaido					
Freshman	19 (11.6)	15 (79.0)	4 (21.1)	<0.0001	8.3 (2.6–26.5)
Other years	145 (88.4)	45 (31.0)	100 (69.0)		
Elsewhere					
Freshman	45 (10.6)	34 (75.6)	11 (24.4)	<0.0001	14.5 (7.0–30.0)
Other years	381 (89.4)	67 (17.6)	314 (82.4)		
**Extracurricular activity**					
Freshman					
Yes	18 (30.0)	9 (50.0)	9 (50.0)	0.01	0.2 (0.1–0.7)
No	42 (70.0)	35 (83.3)	7 (16.7)		
Other years					
Yes	500 (95.1)	111 (22.2)	389 (77.8)	0.02	7.1 (1.0–53.2)
No	26 (4.9)	1 (3.9)	25 (96.2)		
Extracurricular activity (Yes)					
Freshman	18 (3.5)	9 (50.0)	9 (50.0)	0.01	3.5 (1.4–9.0)
Other years	500 (96.5)	111 (22.2)	389 (77.8)		
Extracurricular activity (No)					
Freshman	42 (61.8)	35 (83.3)	7 (16.7)	<0.0001	125 (14.5–1080.9)
Other years	26 (38.2)	1 (3.9)	25 (96.2)		

*p*-value, Fisher’s exact test; * OR = odds ratio of being vaccinated if a student was in the category on the first line vs. the second line; ** Cl = confidence interval using Wald’s method.

**Table 4:** Cross tabulation of vaccination frequency regarding grade/year, medicine and healthcare major, birthplace, and extracurricular activity.

Characteristic	N (%)	Vaccinated (%)	Unvaccinated (%)	*p*-Value	OR * (95% Cl **)
**Freshman**					
Medicine and Healthcare ***					
Freshman	7 (8.9)	6 (85.7)	1 (14.3)	0.04	8.9 (1.0–77.8)
Other faculty	72 (91.1)	29 (40.3)	43 (59.7)		
Other than medicine and healthcare faculty					
Freshman	53 (11.0)	39 (73.6)	14 (26.4)	<0.0001	12.9 (6.7–24.9)
Other faculty	427 (89.0)	76 (17.8)	351 (82.2)		
Freshman					
Medicine and healthcare	7 (11.7)	6 (85.7)	1 (14.3)	0.67	2.2 (0.2–19.5)
Other than medicine and healthcare faculty	53 (88.3)	39 (73.6)	14 (26.4)		
Other than freshman					
Medicine and healthcare	72 (14.4)	29 (40.3)	43 (59.7)	<0.0001	3.1 (1.8–5.3)
Other than medicine and healthcare faculty	427 (85.6)	76 (17.8)	351 (82.2)		
**Place of birth**					
Medicine and healthcare					
Hokkaido	33 (41.8)	16 (48.5)	17 (51.5)	0.65	1.3 (0.5–3.3)
Elsewhere	46 (58.2)	19 (41.3)	27 (58.7)		
Other than medicine and healthcare faculty					
Hokkaido	117 (24.4)	37 (31.6)	80 (68.4)	0.03	1.7 (1.0–2.6)
Elsewhere	363 (75.6)	79 (21.8)	284 (78.2)		
Hokkaido					
Medicine and healthcare	33 (22.0)	16 (48.5)	17 (51.5)	0.10	2.0 (0.9–4.5)
Other faculty	117 (78.0)	37 (31.6)	80 (68.4)		
Elsewhere					
Medicine and healthcare	46 (11.3)	19 (41.3)	27 (58.7)	0.01	2.5 (1.3–4.8)
Other faculty	363 (88.8)	79 (21.8)	284 (78.2)		
**Extracurricular activity**					
Medicine and healthcare					
Yes	69 (89.6)	29 (42.0)	40 (58.0)	0.45	0.4 (0.1–2.0)
No	8 (10.4)	5 (62.5)	3 (37.5)		
Other than medicine and healthcare faculty					
Yes	421 (88.3)	84 (20.0)	337 (80.1)	<0.0001	0.2 (0.1–0.4)
No	56 (11.7)	28 (50.0)	28 (50.0)		
Extracurricular activity (Yes)					
Medicine and healthcare	69 (14.1)	29 (42.0)	40 (58.0)	0.0002	2.9 (1.7–5.0)
Other faculty	421 (85.9)	84 (20.0)	337 (80.1)		
Club activity (No)					
Medicine and healthcare	8 (12.5)	5 (62.5)	3 (37.5)	0.71	1.7 (0.4–7.7)
Other faculty	56 (87.5)	28 (50.0)	28 (50.0)		

*p*-value, Fisher’s exact test; * OR = odds ratio of being vaccinated if a student was in the category on the first line compared with the second line; ** Cl = confidence interval using Wald’s method; *** Faculties of Medicine, Health, Pharmacy, and Dentistry.

**Table 5:** Frequency of vaccination by participants’ attitudes toward influenza and vaccination.

Characteristic	Vaccinated (%)	Unvaccinated (%)	*p*-Value	OR * (95% Cl **)
1. Not worried about influenza	58 (22.9)	195 (77.1)	0.04	0.7 (0.4–1.0)
Otherwise	104 (30.6)	236 (69.4)		
2. Dislike injection or pain with injection	3 (8.6)	32 (91.4)	0.01	0.2 (0.1–0.8)
Otherwise	159 (28.5)	399 (71.5)		
3. Not worried about the clinical seriousness of influenza	12 (14.6)	70 (85.4)	0.0049	0.4 (0.2–0.8)
Otherwise	150 (29.4)	361 (70.7)		
4. Perceived limited effectiveness of vaccination	69 (31.1)	153 (68.9)	0.13	1.3 (0.9–1.9)
Otherwise	93 (25.1)	277 (74.9)		
5. Concerned with the cost of the influenza vaccine	44 (20.3)	173 (79.7)	0.004	0.6 (0.4–0.8)
Otherwise	118 (31.4)	258 (68.6)		
6. Worried about having an allergic reaction	10 (27.8)	26 (72.2)	1.00	1.0 (0.5–2.2)
Otherwise	152 (27.3)	405 (72.7)		
7. Would decide depending on the vaccination status of surrounding people	20 (25.3)	59 (74.7)	0.67	0.9 (0.5–1.5)
Otherwise	142 (27.3)	372 (72.4)		
8. Vaccination depends on having a nearby healthcare facility	31 (30.4)	71 (69.6)	0.46	1.2 (0.8–1.9)
Otherwise	131 (26.7)	360 (73.3)		
9. Worried about adverse reaction following vaccination	22 (33.3)	44 (66.7)	0.24	1.4 (0.8–2.4)
Otherwise	140 (26.6)	387 (73.4)		
10. General health status	40 (28.8)	99 (71.2)	0.66	1.1 (0.7–1.7)
Otherwise	122 (26.9)	332 (73.1)		
11. Encouragement from parents	31 (30.1)	72 (69.9)	0.54	1.2 (0.7–1.9)
Otherwise	131 (26.7)	359 (73.3)		
12. Vaccination depends on available time	48 (23.9)	153 (76.1)	0.21	0.8 (0.5–1.1)
Otherwise	114 (29.1)	278 (70.9)		

* OR = odds ratio of being vaccinated if a student was in the category on the first line compared with the second line; ** Cl = confidence interval using Wald’s method.

## Supplementary Materials

The following are available online at https://www.mdpi.com/2076-393X/8/1/52/s1, Table S1: English translation of the questionnaire.
