# Supplementary material for: Exploring Influenza Vaccine Uptake and Its Determinants among University Students: A Cross-Sectional Study
Source: Vaccines (Basel). 2020 Jan 28;8(1):52. doi: 10.3390/vaccines8010052 (PMC7158679; doi:10.3390/vaccines8010052)
Supplement: Supplementary file 1 [file vaccines-08-00052-s001.pdf]

No.

mm / dd / 2019

Please answer to all questions below, filling in the blank space or giving a circle to what it applies to you.

1. Your demographic information.

Faculty ： Grade

Sex： male ・ female Age

Birth place : Hokkaido ・ Others

Parents’ education: Father(High school university graduate school vocational school others

Mother (High school university graduate school vocational school others

Allowance from family: ： Yes ・ No

Scholarship(have a plan to receive) ： Yes ・ No

Club activities Yes ・ No

2. Did you receive the flu vaccine last season (July 2018~ March 2019) Yes ・ No

3. Please select all factors that lead you to receive vaccination:

・Not worried about influenza

・Dislike injection or its pain

・Perceived limited effectiveness of vaccination

・Not worried about clinical seriousness of influenza

・Concerned with the cost of influenza vaccine

・Having any allergic reaction

・Would decide depending on vaccination status of surrounding people

・Vaccination depends on whether I know nearby healthcare facility

・General health appearance

・Worried about adverse reaction of vaccination

・Encouragement from parents

・Vaccination depends on available time to undertake vaccination
